# MicroRNAs, polyamines, and the activities antioxidant enzymes are associated with in vitro rooting in white pine (*Pinus strobus* L.)

**DOI:** 10.1186/s40064-016-2080-1

**Published:** 2016-04-06

**Authors:** Yunjun Fei, Bo Xiao, Man Yang, Qiong Ding, Wei Tang

**Affiliations:** College of Horticulture and Gardening, Yangtze University, Jingzhou, 434025 Hubei China; Institute for Genome Sciences and Policy, Duke University, Durham, NC 27708 USA

**Keywords:** Antioxidant enzyme, MicroRNAs, *Pinus strobus*, Polyamines, Root formation

## Abstract

Molecular mechanism of in vitro rooting in conifer is not fully understood. After establishment of a regeneration procedure in eastern white pine (*Pinus strobus L.*) using mature embryos as explants to induce shoot formation on medium containing 3 μM IAA, 6 μM BA and 6 μM TDZ and induce root formation on medium containing 0.001-0.05 μM IAA, 0.001–0.05 μM IBA, 0.001–0.05 μM TDZ, we have investigated the changes of polyamine content and the activities of antioxidant enzymes during in vitro rooting in *P. strobus*. Our results demonstrated that putrescine (Put), spermidine (Spd), and spermine (Spm) did not increase in *P. strobus* during the first week of rooting on medium supplemented with 0.01 μM indole-3-acetic acid (IAA), whereas the levels of Put, Spd, and Spm increased during the 1st–3rd week of culture on medium with IAA, and then decreased on medium with IAA. No such a change in Put, Spd, and Spm was observed on medium without IAA. Measurement of antioxidant enzyme activity demonstrated that the activities of polyphenol oxidase, catalase, and peroxidase slightly increased in the first week of culture and reached to the highest peak in the 3rd–5th week of culture. Quantitative RT-PCR results indicated that miR160 was increased on the 7th day, miR162, miR397, and miR408 was increased from the 21th to 35th day, miR857 was increased on the 35th day, and miR827 was increased on the 49th day. These results demonstrated that enhanced polyamine biosynthesis, antioxidant enzyme activity, and microRNAs are correlated with the root induction and formation in *P. strobus*.

## Background

In vitro plant regeneration has facilitated large-scale propagation of plants and provides a number of opportunities for genetic engineering of economically important crops (Gaspar et al. 1996; Kintzios et al. 2004; Rout et al. 2000; Thomas et al. 2004) and forest tree species (Attree and Fowke 1993; Klimaszewska et al. 2001b; Schwarz et al. 1988; Stojicic et al. 2012; Tang and Newton 2003). Investigation of in vitro processes in many plant species has been focused on the influences of the plant genotype, plant hormones, and physiological stages of cultured tissues (Rout et al. 2000; Thomas et al. 2004). In many important agricultural plants and trees, the low efficiency of in vitro rooting is a major impediment to the high frequency in vitro regeneration for their improvement via gene transfer (Blando et al. 2013; Macedo et al. 2013; Sediva et al. 2013; Tang and Newton 2003; Wiszniewska et al. 2013). Therefore, it is important to investigate factors affecting in vitro regeneration, especially rooting in conifers (Attree and Fowke 1993; Gaspar et al. 1996; Rout et al. 2000).

Rooting is a unique way of organogenesis in plant (Valdes et al. 2001). The ability of rooting depends on the plant species and the culture condition (Devi et al. 2013; Krishna et al. 2013; Mendes et al. 2011). Understanding how somatic cells differentiate into organ is very important for us to investigate plant development (Couee et al. 2004; Mendes et al. 2011; Valdes et al. 2001). Protein synthesis and the related metabolic pathways are related to organogenesis (Jarvis et al. 1985; Tantikanjana et al. 2001; Thomas et al. 2004; Torelli et al. 2004; Trupiano et al. 2013). The activities of antioxidant enzymes have been investigated during organ formation (Mitrovic et al. 2012). During the process of callus differentiation, the activity of SOD increased in the early stages of culture and then decreased, and the activity of CAT declined while the activity of POD gradually increased (Dobnik et al. 2013; Mitrovic et al. 2012; Mytinova et al. 2010; Tang and Newton 2005a; Vatankhah et al. 2010). These results suggested that the activities of antioxidant enzymes were related to the process of differentiation in plants, and may serve as biological markers in the process of organ primordial formation (Devi et al. 2013; Mitrovic et al. 2012; Mytinova et al. 2010; Tang and Newton 2005a; Vatankhah et al. 2010).

MicroRNAs are endogenous noncoding RNAs that act as important regulators of gene expression and regulate various developmental processes by targeting genes encode transcription factors in plants (Gutierrez et al. 2009). MicroRNAs are now recognized as regulators of processes related to root formation including the regulation of transcription factors, nutrient uptake, stress signaling, and growth signaling (Tang and Tang 2016). MicroRNAs regulate plant root formation by targeting different auxin response factors. For example, the targets of the microRNA miR167, auxin response factors ARF6 and ARF8, are positive regulators of root formation and the target of miR160, ARF17, is a negative regulator (Gutierrez et al. 2012; Mallory et al. 2005). However, changes of polyamine biosynthesis, the activities of antioxidant enzymes, and the levels of microRNAs, have not been investigated in conifers during in vitro rooting.

Eastern white pine (*Pinus strobus* L.) is an important softwood and Christmas trees species in the area of the Appalachians. In vitro regeneration of *P. strobus* has been reported (Flinn et al. 1988; Kaul 1987; Schwarz et al. 1988; Webb et al. 1988). However, the frequency of plant regeneration was relatively low because of the difficulty in rooting. Although in vitro plant regeneration in *P. strobus* has been improved, it is still not applicable in tree improvement programs (Garin et al. 2000; Klimaszewska et al. 2000, 2001a, b). We previously reported in vitro plant regeneration from mature embryos of *P. strobus* (Tang and Newton 2005a, b). In this investigation, our studies have focused on polyamine biosynthesis, antioxidant enzyme activity, and expression of microRNAs during in vitro rooting in *P. strobus*. We examined the rooting frequency of shoots derived from genotypes 1-721, 2-007, 3-011, and 3-101, the levels of polyamine biosynthesis, and activities of antioxidant enzymes during in vitro rooting. Results of our study should be helpful to elucidate the involvement of polyamine biosynthesis, the activities of antioxidant enzymes, and the expression of microRNAs during in vitro root formation in *P. strobus*.

## Results

### Induction of shoots

Induction of shoots in eastern white pine was performed by cultured mature embryos induction medium with 5 μM IAA, 3 μM IBA, and 3 μM BA for 7 days, then embryos were transferred onto medium containing 3 μM IAA, 6 μM BA and 6 μM TDZ for 14 days. Shoot formation was observed in the 3–5 weeks (on medium with 3 μM BA, 3 μM Kinetin, and 6 μM TDZ) after mature embryos were cultured on medium with IAA, IBA, and BA for 7 days, then cultured on medium with IAA, IBA, and TDZ for 14 days. Shoot formation was not obtained from mature embryos that were cultured on medium without IAA, IBA, BA, and any other plant growth regulator for 7 days (as a control), then cultured on medium with IAA, IBA, and TDZ for 14 days. Shoot formation was also not obtained from mature embryos that cultured on medium with IAA, IBA, and BA for 7 days, then cultured on medium with IAA, IBA, and without TDZ for 14 days. Shoot formation was obtained only on medium with TDZ. After shoot formation was induced, different concentrations of casein enzymatic hydrolysate (CH) and glutamine were tested, respectively. Our results showed that higher frequency of shoot regeneration was obtained on medium with 500–600 mg l^−1^ CH and 600–700 mg l^−1^ glutamine, compared to control. These results are consistent with our previous findings in *P. strobus* (Tang and Newton 2005a, b).

### Rooting of shoots and plant regeneration

**Fig. 1:**
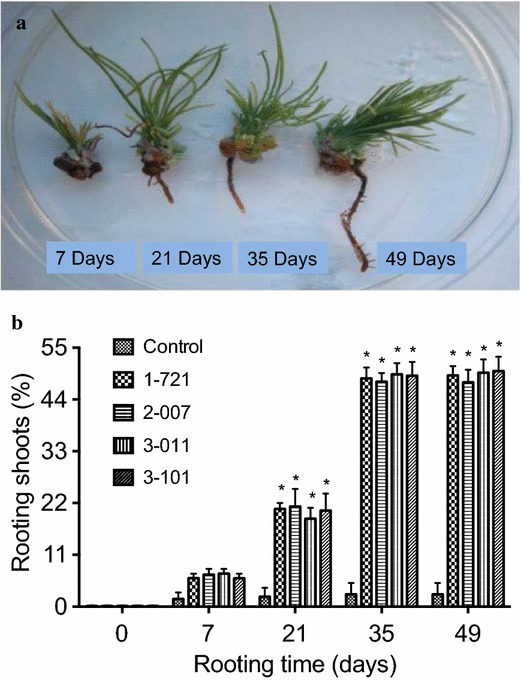
In vitro rooting of *P. strobus.* **a** Root formation from elongated adventitious shoots at different time points of culture on media with 0.01 μM IAA. Roots formed from elongated adventitious shoots were shown at 7, 21, 35, and 49 days of culture. **b** Rooting rate of shoots derived from genotypes 1-721, 2-007, 3-011, and 3-101 on medium containing 0.01 μM IAA. Each treatment was replicated five times, and each replicate consisted of 30–50 shoots. Values represent the mean ± SD. Values followed by *one asterisk* are significantly different from the corresponding control value (P < 0.05; n = 5) by ANOVA

Elongated adventitious shoots were cultured on media with 0.01 μM IAA at different time points of culture for root formation and roots formed from shoots were obtained at 7, 21, 35, and 49 days of culture (Fig. 1a). On medium supplemented with more than 1 μM IAA, no rooting was observed (data are not presented). Root formation was obtained on medium containing 0.001–0.05 μM IAA (Table 1). The highest rooting percentage was obtained at 49 days of culture on medium supplemented with 0.01 μM IAA (with the exception of 2-007), with no statistical differences from 35 days (Fig. 1b). After shoots were cultured on medium with 0.01 μM IAA for 35 days, well-developed roots were obtained (Fig. 1a). Influence of different plant growth regulators on in vitro rooting was examined on medium supplemented with different concentrations of IAA, IBA, and TDZ (Tables 1, 2, 3). Among different concentrations of IAA, IBA, or TDZ tested, higher frequency of rooting was obtained on medium supplemented with 0.05 μM IAA, 0.05 μM IBA, or 0.01 μM TDZ (Tables 1, 2, 3). In this study, 668 plantlets were regenerated from 900 shoots and these plantlets were established in soil.

**Table 1:** Effect of IAA on the percentage of rooting shoots in *P. strobus*

Genotypes	Rooting shoots (%)				
	0	0.001 μM	0.005 μM	0.01 μM	0.05 μM
1-721	3.9 ± 1.1a	13.4 ± 1.8b	26.5 ± 2.7b	46.8 ± 3.8b	49.1 ± 3.8b
2-007	3.4 ± 1.6a	16.1 ± 2.7b	29.5 ± 2.9b	49.6 ± 3.9b	52.3 ± 3.6b
3-011	3.6 ± 1.3a	15.5 ± 1.9b	27.3 ± 2.5b	48.7 ± 3.8b	49.5 ± 3.7b
3-101	3.1 ± 1.2a	14.4 ± 1.8b	26.8 ± 2.8b	47.6 ± 3.7b	48.1 ± 3.9b

Percentage of shoot rooting were measured 35 days after shoots were transferred into medium containing 0.001, 0.005, 0.01, or 0.05 μM IAA, respectively. Each experiment was replicated five times, and each replicate consisted of 30–50 shoots. Values represent the mean ± SD. Values followed by different letters are significantly different (α = 0.05) by ANOVA

**Table 2:** Effect of IBA on the percentage of rooting shoots in *P. strobus*

Genotypes	Rooting shoots (%)				
	0	0.001 μM	0.005 μM	0.01 μM	0.05 μM
1-721	3.7 ± 1.8a	6.4 ± 1.7a	19.9 ± 2.9b	25.4 ± 2.9b	27.7 ± 3.6b
2-007	3.9 ± 1.6a	6.7 ± 1.6a	18.8 ± 2.7b	28.7 ± 2.7b	30.9 ± 3.7b
3-011	3.8 ± 1.7a	5.6 ± 1.5a	19.6 ± 2.6b	29.6 ± 2.8b	31.8 ± 3.6b
3-101	3.7 ± 1.5a	6.8 ± 1.9a	17.9 ± 2.8b	26.8 ± 2.9b	29.7 ± 3.5b

Percentage of shoot rooting were measured 35 days after shoots were transferred into medium containing 0.001, 0.005, 0.01, or 0.05 μM IBA, respectively. Each experiment was replicated five times, and each replicate consisted of 30–50 shoots. Values represent the mean ± SD. Values followed by different letters are significantly different (α = 0.05) by ANOVA

**Table 3:** Effect of TDZ on the percentage of rooting shoots in *P. strobus*

Genotypes	Rooting shoots (%)				
	0	0.001 μM	0.005 μM	0.01 μM	0.05 μM
1-721	3.5 ± 1.1a	4.4 ± 1.7a	5.4 ± 1.7a	43.5 ± 3.9b	28.6 ± 2.6b
2-007	3.2 ± 1.4a	5.4 ± 1.8a	6.7 ± 1.9a	44.3 ± 3.5b	23.4 ± 2.8b
3-011	3.1 ± 1.3a	5.3 ± 1.6a	6.6 ± 1.8a	41.4 ± 3.8b	27.5 ± 2.7b
3-101	3.3 ± 1.6a	5.2 ± 1.3a	6.5 ± 1.3a	42.5 ± 3.4b	29.7 ± 2.5b

Percentage of shoot rooting were measured 35 days after shoots were transferred into media containing 0.001, 0.005, 0.01, or 0.05 μM TDZ, respectively. Each experiment was replicated five times, and each replicate consisted of 30–50 shoots. Values represent the mean ± SD. Values followed by different letters are significantly different (α = 0.05) by ANOVA

### Polyamines synthesis during in vitro rooting

**Fig. 2:**
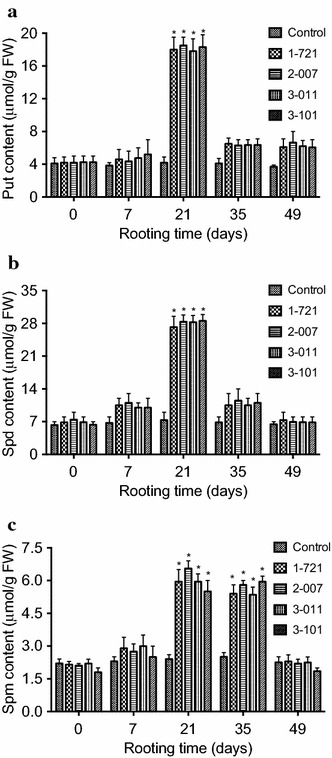
Changes of polyamines **a** putrescine (Put), **b** spermidine (Spd), and **c** spermine (Spm) levels during rooting. Polyamines contents were measured 7, 21, 35, and 49 days after shoots were transferred into media containing 0.01 μM IAA. Shoots for the control were derived from genotype 1-721 and were transferred to media without IAA. Each experiment was replicated five times, and each replicate consisted of 6–8 samples. Values represent the mean ± SD. Values followed by *one asterisk* are significantly different from the corresponding control value (P < 0.05; n = 5) by ANOVA

To determine whether changes of the endogenous polyamines is associated with in vitro rooting in eastern white pine, the amount of putrescine (Put), spermidine (Spd), and spermine (Spm) were measured 7, 21, 35, and 49 days after shoots were cultured on media supplemented with 0.01 μM indole-3-acetic acid (IAA) in *P. strobus*. Our results showed that the amount of Put, Spd, and Spm was not affected during the first week of rooting on medium containing 0.01 μM IAA (Fig. 2). In contrast, marked changes were found on the amount of Put, Spd, and Spm in the 3rd–5th week of culture on medium with IAA (Fig. 2).

Compared to the control, the Put contents increased 445.7–468.6 % in the 3rd week of rooting among different genotypes 1-721, 2-007, 3-011, and 3-101. In the 7th week of rooting, the Put contents increased only 57.1–68.6 % (Fig. 2). Compared to the control, the Spd contents increased 417.5–424.6 % in the 3rd week of rooting among different genotypes 1-721, 2-007, 3-011, and 3-101 (Fig. 2). Compared to the control, the Spm contents increased 332–344 % in the 3rd week of rooting among different genotypes 1-721, 2-007, 3-011, and 3-101 (Fig. 2). In the 7th week of rooting, the Spd and Spm contents backed to the levels in the first week of in vitro rooting (Fig. 2).

Synthesis of Put, Spd, and Spm were increased from the 3rd to 5th week during in vitro rooting. Amount of Put, Spd, and Spm were decreased from the 5th to 7th week on medium supplemented with IAA. No similar change in Put, Spd, and Spm was obtained on medium without IAA. These results indicate that altered polyamine metabolism is associated with in vitro rooting and imply that the change in the polyamine amounts during in vitro rooting could be related to in vitro root induction and formation.

### Activities of antioxidant enzymes during in vitro rooting

**Fig. 3:**
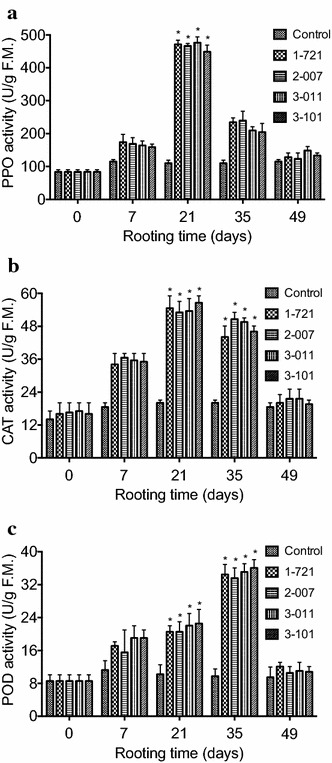
Changes of antioxidant enzyme activity during rooting. Activities of **a** PPO, **b** CAT, and **c** POD were measured 7, 21, 35, and 49 days after shoots were transferred into media containing 0.01 μM IAA. Shoots for the control were derived from genotype 1-721 and were transferred to media without IAA. Each experiment was replicated five times, and each replicate consisted of 6–8 samples. Values represent the mean ± SD. Values followed by *one asterisk* are significantly different from the corresponding control value (P < 0.05; n = 5) by ANOVA

To determine whether changes in antioxidant enzymes are associated with in vitro rooting in eastern white pine, the levels of PPO, CAT, and POD were determined 7, 21, 35, and 49 days after shoots were cultured on media supplemented with 0.01 μM indole-3-acetic acid (IAA) during in vitro rooting in *P. strobus*. Our results showed that the levels of PPO, CAT, and POD were largely unaffected during the first week of rooting on medium supplemented with 0.01 μM IAA (Fig. 3). In contrast, marked changes were identified in the levels of PPO, CAT, and POD in the 3rd–5th week of culture on medium with IAA (Fig. 3). The highest levels of PPO and CAT activities were obtained in the 3rd week of culture on rooting medium containing 0.01 μM IAA, while POD activity increased in the 5th week (Fig. 3).

### Expression of microRNAs during in vitro rooting

**Fig. 4:**
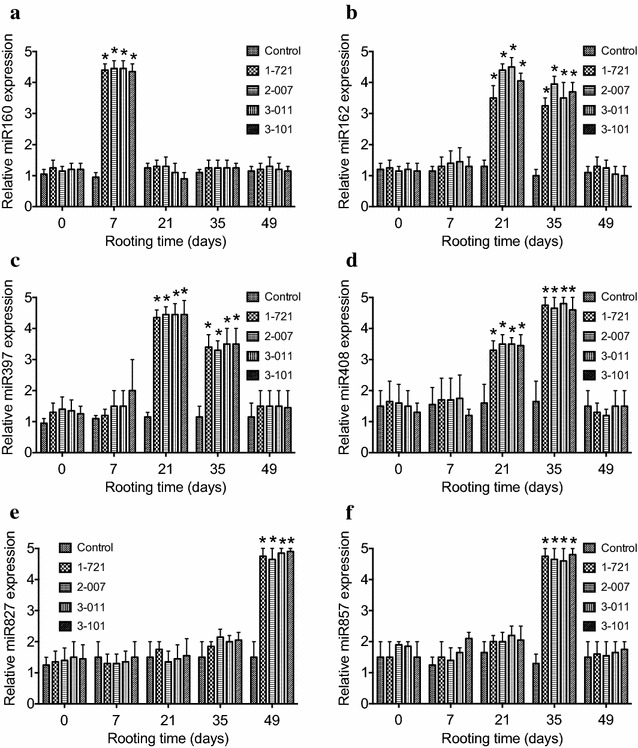
Expression of miRNAs during the time course of in vitro rooting in *P. strobus*. RNA was isolated from seedlings at different stages (7, 21, 35, 49 days) of rooting after shoots were transferred into media containing 0.01 μM IAA. Shoots for the control were derived from genotype 1-721 and were transferred to media without IAA. Relative expression was indicated by the log2 value. Values followed by *one asterisk* are significantly different from the corresponding control value (P < 0.05; n = 3) by ANOVA

To determine whether changes in expression of microRNAs are associated with in vitro rooting in eastern white pine, the levels of miR160, miR162, miR397, miR408, miR827, and miR857 were examined on the 7th, 21th, 35th, and 49th day during in vitro rooting in *P. strobus*. Our quantitative RT-PCR results demonstrated that miR160 was increased on the 7th day during the time course of in vitro rooting and then decreased, whereas miR162, miR397, and miR408 were increased from the 21th to 35th day before return to the control level, miR827 was increased on the 49th day and miR857 was increased on the 35th day (Fig. 4). These results suggest microRNAs may play important roles in regulating plant root initiation and development, as well as in modulating their root systems.

## Discussion

Plant tissue culture based plant regeneration system is important for improving conifer properties through genetic transformation (Attree and Fowke 1993; Kaul 1987; Tang and Newton 2003, 2005a, b, c; Tang et al. 1998). Plant growth regulators play important roles in in vitro regeneration (Ahkami et al. 2013; Correa et al. 2012; Jung et al. 2012; Kaul 1987; Okazaki and Saito 2012). Although a plant regeneration protocol via in vitro shoot organogenesis has been established in *P. strobus* (Kaul 1987; Tang and Newton 2005a, b), the frequency of in vitro rooting was relative low and development of high frequency in vitro rooting procedure from adventitious shoots was needed. Kaul (1987) has reported the use of auxin (NAA) for root induction after successful induction of shoots (Kaul 1987). In our investigation, we found that NAA also induce callus formation even at low concentration in *P. strobus.* We have examined the effects of different plant growth regulators including IAA, IBA, and TDZ on in vitro rooting in *P. strobus*. Our results demonstrated that the higher rooting rate was induced on medium containing 0.01 μM IAA (Tables 1), compared to medium containing IBA or TDZ (Tables 2, 3). Another advantage using 0.01 μM IAA for rooting is not inducing callus formation but promote root elongation and growth.

Expression of different genes regulates in vitro root morphogenesis and identification of specific genes expressed or gene products at different morphogenetic stages will increase our understanding of molecular mechanisms in plants (Burritt and Leung 1996; Torelli et al. 2004). Expression of a putative serine-threonine kinase (LESK1) has been identified as a marker of in vitro caulogenesis in tomato (Torelli et al. 2004). It has been reported that correlations between changes in endogenous polyamines (putrescine) and hormones (auxins) are critical to in vitro root regeneration (Arigita et al. 2004; Mendes et al. 2011; Mytinova et al. 2010; Romano et al. 1991; Tantikanjana et al. 2001). Among different polyamines we have examined in the present study, we found that the content of Put and Spd increased more than fourfolds at 21 days of rooting among different genotypes 1-721, 2-007, 3-011, and 3-101, compared to the control (Fig. 2a, b). We also found that the content of Spm increased more than three folds from 21 to 35 days of rooting, compared to the control (Fig. 2c). We speculate that increased endogenous Put and Spd content may promote root initiation and increased endogenous Spm content may induce root initiation and growth because no such a change in the levels of Put, Spd, and Spm was obtained from the control shoots. Our results indicate that altered polyamine metabolism is associated with in vitro rooting and that the changes in polyamines content during in vitro rooting could be related to in vitro root induction and formation. These results may be of value for further application of in vitro regeneration and genetic transformation in *P. strobus*.

Previous studies showed that the activities of antioxidant enzymes were associated with in vitro organogenesis in plants (Mitrovic et al. 2012). For example, in the early shoot regeneration culture, SOD activity increased and CAT activity decreased, while POD gradually increased after differentiation (Devi et al. 2013; Mitrovic et al. 2012; Mytinova et al. 2010; Vatankhah et al. 2010). We have examined the PPO, CAT, and POD activities during in vitro rooting in *P. strobus*. Our results demonstrated that the highest levels of PPO and CAT were obtained at 21 days of culture on rooting medium (Fig. 3a, b) and that the highest levels of POD was obtained at 35 days of culture on rooting medium (Fig. 3c). Because significant increase of Put, Spd, and Spm was also observed at 21 days of culture (Fig. 2), we speculate that enhanced polyamine biosynthesis may cooperate with antioxidant enzyme activity to promote the root induction and formation in *P. strobus* in a time-dependent manner. However, how this cooperation regulates root initiation and formation in *P. strobus* remains to be determined.

It has been reported that the activity of antioxidant enzyme reflects the oxidative stage of the root and reactive oxygen species (ROS) play a diversity of roles in root growth and development in a number of plant model systems (Carol and Dolan 2006; Knight 2007; Kwasniewski et al. 2013). ROS are required for cell expansion during the morphogenesis of roots and for root hair growth where they control the activity of calcium channels required for polar growth (Carol and Dolan 2006; Knight 2007). For example, the function of NADPH oxidase-related ROS in root hair development has been reported extensively in studies on Arabidopsis (Kwasniewski et al. 2013), indicating that the role of ROS in the control of root hair growth may provide insight into the mechanism of plant cell growth in general (Carol and Dolan 2006; Knight 2007). In this investigation, significant increase of the PPO, CAT, and POD activities was obtained during in vitro rooting, suggesting that the PPO, CAT, and POD-related ROS may contribute to root initiation, growth, and development in *P. strobus*.

Root initiation and development are not only associated with changes in endogenous polyamines and the activities of antioxidant enzymes, but also closely linked with expression levels of microRNAs (Fig. 4). It has been reported that miRNAs modulate the plant root system to adapt the fluctuation of nutrient availability (Meng et al. 2010). Increased level of miR395 resulted in advantageous root growth (Liang et al. 2015). Reduction in miR167 plays a role in the balance between lateral root initiation and emergence (Gifford et al. 2008). Increased expression of miR399b promoted later root initiation and inhibited primary root growth (Yanik et al. 2013; Zhang et al. 2009). Increased miR160 regulates root cap formation and primary root length (Meng et al. 2010). Although many miRNAs were differentially expressed during the phases of root initiation, development, and growth, some miRNAs were specifically related to specific stages of in vitro rooting. We observed that miR162, miR397, and miR408 was increased from the 21st to 35th day, whereas miR160, miR857, and miR827 showed the largest changes on the time points of 7th, 35th, and 49th day during in vitro rooting, respectively. Our studies suggested that miR160 regulates the root initiation, miR827 mediates root growth, and miR162, miR397, miR408, and miR857 play a particular role in the development and elongation of roots. Recently, It has been reported that decreased expression of miR160 changed the root systems and increased expression of miR160 facilitated root growth (Liang et al. 2015; Yanik et al. 2013). Correspondingly, miR160 was induced on the 7th day of rooting in *P. strobus*, demonstrating that miR160 directly affects the root initiation.

Although our experimental results demonstrate that microRNAs, polyamines, and antioxidant enzymes are involved in the process of rooting in *P. strobus*, detailed mechanisms on how these factors regulate root initiation and formation remains to be elucidated. Considering significant increase of miR162, miR397, and miR408 expression were observed at 21 days of culture and contents of Put, Spd, and Spm, as well as the activities of antioxidant enzymes PPO, CAT, and POD are significantly increased at the same time, we speculate that miR162, miR397, and miR408 may target the negative regulators of Put, Spd, Spm, PPO, CAT, and POD biosynthesis in *P. strobus.* This kind of regulation for Spm, CAT, and POD biosynthesis may be extended to the 35th day of culture because significant increase of miR162, miR397, and miR408 expression are consistent with the significant increase of Spm, CAT, and POD biosynthesis at this stage. In addition, miR857 may be an additional regulator that targets the negative regulators of Spm, CAT, and POD biosynthesis at 35 days of culture. Significant increase of miR160 expression and miR827 expression were obtained at 7 and 49 days of culture, respectively, but no significant changes of Put, Spd, Spm, PPO, CAT, and POD biosynthesis were observed at the same stage, demonstrating that miR160 and miR827 may regulate other factors that are not related to Put, Spd, Spm, PPO, CAT, and POD biosynthesis but contribute to root initiation and formation in *P. strobus*. Our findings should be valuable to elucidate the involvement of polyamine biosynthesis and antioxidant enzyme activity during in vitro root formation in *P. strobus*.

## Conclusions

Based on the experimental results obtained in this investigation, we speculate that expression levels of microRNAs, the changes of polyamine content, and the activities of antioxidant enzymes may be involved in molecular mechanisms of in vitro rooting in *P. strobus*. Measurement of the contents of polyamines, the activities of antioxidant enzymes, and the levels of microRNAs expression demonstrated that miR162, miR397, miR408, and miR857 may regulate root initiation and formation by targeting the negative regulators of Put, Spd, Spm, PPO, CAT, and POD biosynthesis and miR160 and miR827 may regulate root initiation and formation by targeting other factors that are not related to Put, Spd, Spm, PPO, CAT, and POD biosynthesis but affect root initiation and formation in *P. strobus*. These results indicate that polyamines biosynthesis, antioxidant enzyme activity, and microRNAs expression are correlated to the root induction and formation in a time-dependent manner in *P. strobus*.

## Methods

### Plant material

Dry seeds (1-721, 2-007, 3-011, and 3-101) of eastern white pine (*Pinus strobus* L.) were collected from tree breeding program and purchased from F. W. Schumacher Co., Inc., Sandwich, MA 02563, USA. After thoroughly washing, seeds were disinfected by following the procedure previously described (Tang et al. 1998). Mature zygotic embryos were taken out from the seeds and cultured on callus induction medium.

### Adventitious shoots induction and rooting

Mature embryos were cultured on TE medium (Tang and Newton 2005a, b; Tang et al. 1998) supplemented with 5 μM indole-3-acetic acid (IAA), 3 μM N6-benzyladenine (BA), and 3 μM IBA for 1 week. Embryos were transferred onto induction medium [containing 6 μM BA, 3 μM IAA, and 6 μM Thidiazuron (TDZ)] for 2 weeks (Tang and Newton 2005a, b). Regeneration of adventitious shoots was conducted as previously reported (Tang and Newton 2005a, b).

Shoots from TE medium supplemented with 2 μM IAA and 1 μM BA, 1.5 to 3 cm in height, were transferred onto medium supplemented with 0.001–0.05 μM of IAA, IBA, or TDZ and 0.5 μM BA for root formation. The frequency of rooting was determined in the 7th, 21th, 35th, and 49th days of culture. Rooting was maintained at 25 °C in the light (50 μmol m^−2^s^−1^, 16-hphotoperiod). Plantlets were transferred to containers containing a perlite: peatmoss: vermicu-lite (1:1:1 v/v/v) mixture and grown in a greenhouse (Tang and Newton 2005a, b) until tissues are taken for different assays.

### Determination of polyamines

Determination of polyamines putrescine (Put), spermidine (Spd), and spermine (Spm) from tissues of *P. strobus* was carried out as described previously (Flores and Galston 1982a, b). Samples were examined using a HPLC and Spector Monitor 3200 Detector by following the manual of the facility (Tang and Newton 2005c). The measured polyamines are total PAs.

### Measurement of antioxidant enzyme activity

Activity of antioxidant enzymes PPO was measured by following the modified methods previously described (Ruuhola et al. 2011, 2013). The activity of CAT (EC 1.11.1.6) was determined spectrophotometrically as previously described (Beers and Sizer 1952). The decomposition of 1 mmol H_2_O_2_ per gram FW in 1 min was defined as one unit. The activity of POD (EC 1.11.1.7) was determined according to the procedure of Kim and Yoo (1996), with a Shimadzu UV-120IV spectrophotometer. The amount of POD catalyzing the oxidation of 1 mol guaiacol in 1 min was defined as one unit (Tang and Newton 2005a).

### Total RNA isolation, cDNA library construction, and quantitative real-time PCR (qPCR)

Total RNA was isolated from frozen pine tissues using TRIzol reagent according to the manufacturer’s protocol (Invitrogen). The high quality cDNA were prepared using a TaqMan^®^ MicroRNA Reverse Transcription Kit (Applied Biosystems). For miRNA expression analysis, TaqMan^®^ MicroRNA Assays (Applied Biosystems) are carried out to amplify RNAs for quantitation of miRNAs. The controls used were as suggested by the manufacturer’s manual. The *U6* gene was used as an internal control. Samples were examined in triplicate on the Applied Biosystems 7900HT System, according to the manufacturer’s manual. Two specific primers were used to amplify each of miRNAs. Primers used for Real-Time PCR are listed in Table 4.

**Table 4:** Primers used for amplification of microRNA expression

miRNA name	Primer sequence (5′–3′)
miR160	R: GTCGTATCCAGTGCAGGGTCCGAGGTATTCGCACTGGATACGACTGGCATA
	F: GCATGCTGCCTGGCTCCCTGT
miR162	R: AGTGGTTTATCGATCTCTTCCTTG
	F: GTGGTTCAAGCGTTTTATTGTTG
miR397	R: GTCGTATCCAGTGCAGGGTCCGAGGTATTCGCACTGGATACGACCATCAA
	F: GCGAGCTCATTGAGTGCAGCG
miR408	R: GTCGTATCCAGTGCAGGGTCCGAGGTATTCGCACTGGATACGACCATGCT
	F: GTCAGCACAGGGAACAAGCAG
miR827	R: GTCGTATCCAGTGCAGGGTCCGAGGTATTCGCACTGGATACGACAGTTTG
	F: GGCGCGUUAGAUGACCAUCAA
miR857	R: GTCGTATCCAGTGCAGGGTCCGAGGTATTCGCACTGGATACGACATACAC
	F: GCGGCGTTTTGTATGTTGAAG

### Statistical analyses

Analysis of experimental data was performed as previously described (Tang and Newton 2005a) using the General Linear Model procedure of SAS (SAS, Cary, N.C.), employing ANOVA. Significant differences between mean values were determined with the Least Significant Difference test at 5 % level of probability.

## Abbreviations

BA: N6-benzyladenine
CAT: catalase
CH: casein enzymatic hydrolysate
IAA: indole-3-acetic acid
IBA: indole-3-butyric acid
KIN: kinetin
POD: peroxidase
PPO: polyphenol oxidase
Put: putrescine
Spd: spermidine
Spm: spermine
TDZ: thidiazuron
